# Cruciate ligament force of knees following mobile-bearing unicompartmental knee arthroplasty is larger than the preoperative value

**DOI:** 10.1038/s41598-021-97655-z

**Published:** 2021-09-14

**Authors:** Kenichi Kono, Hiroshi Inui, Tetsuya Tomita, Darryl D. D’Lima, Takaharu Yamazaki, Shoji Konda, Shuji Taketomi, Ryota Yamagami, Kohei Kawaguchi, Shin Sameshima, Tomofumi Kage, Sakae Tanaka

**Affiliations:** 1 Department of Orthopaedic Surgery, Faculty of Medicine, The University of Tokyo, 7-3-1 Hongo, Bunkyo-ku, Tokyo, 113-0033 Japan; 2 Department of Orthopaedic Biomaterial Science, Osaka University Graduate School of Medicine, 2-2 Yamada-oka, Suita, Osaka 565-0871 Japan; 3 Department of Molecular Medicine Arthritis Research, The Scripps Research Institute, La Jolla, CA 92037 USA; 4 Shiley Center for Orthopaedic Research & Education at Scripps Clinic, La Jolla, CA 92121 USA; 5 Department of Information Systems, Faculty of Engineering, Saitama Institute of Technology, 1690 Fusaiji, Fukaya, Saitama 369-0293 Japan; 6 Department of Health and Sport Sciences, Osaka University Graduate School of Medicine, 2-2 Yamada-oka, Suita, Osaka 565-0871 Japan

**Keywords:** Medical research, Engineering

## Abstract

We analyzed the implantation effects on cruciate ligament force in unicompartmental knee arthroplasty (UKA) and determined whether kinematics is associated with the cruciate ligament force. We examined 16 patients (17 knees) undergoing medial UKA. Under fluoroscopy, each participant performed a deep knee bend before and after UKA. A two-dimensional/three-dimensional registration technique was employed to measure tibiofemoral kinematics. Forces in the anteromedial and posterolateral bundles of both the anterior cruciate ligament (aACL and pACL) and the anterolateral and posteromedial bundles of the posterior cruciate ligament (aPCL and pPCL) during knee flexion were analyzed pre- and post-UKA. Correlations between changes in kinematics and ligament forces post-UKA were also analyzed. Preoperatively, the aACL forces were highly correlated with anteroposterior (AP) translation of the lateral condyles (Correlation coefficient [r] = 0.59). The pPCL forces were highly correlated with the varus–valgus angulation (r =  − 0.57). However, postoperatively, the PCL forces in both bundles were highly correlated with the AP translation of the medial femoral condyle (aPCL: r = 0.62, pPCL: r = 0.60). The ACL and PCL forces of the knees post-UKA were larger than those of the knees pre-UKA. Kinematic changes were significantly correlated with the cruciate ligament force changes.

## Introduction

Unicompartmental knee arthroplasty (UKA) is a procedure that is associated with a high level of patient satisfaction among patients with osteoarthritis (OA). Several studies have reported that more patients are satisfied with UKA than with total knee arthroplasty (TKA)^1,2^. In addition, some studies have demonstrated a greater rate of return to sports activities after UKA than after TKA^3,4^. The knee kinematics observed following UKA have been shown to be closer to normal than those following bicruciate-retaining TKA (BCR-TKA)^5^. Furthermore, the kinematics following UKA generally remain similar to preoperative knee kinematics^6,7^, except for the varus–valgus angulation^6^. A previous study has reported that preoperative and postoperative femurs displayed external rotation with flexion, and that the anteroposterior (AP) translation of the medial and lateral sides indicated posterior movement with flexion^6^. The difference between the medial and lateral sides of both preoperative and postoperative knees following UKA represented a medial pivot pattern from 0° to 50° of flexion and from 50° to 130° of flexion, with observed posterior rollback^6^. There were no significant differences in the femoral rotation angles, AP translations, and kinematic pathways in the mid-flexion range of motion before and after UKA^6^. In contrast, preoperative UKA knees showed a significant varus alignment from 10° to 60° of flexion, compared with postoperative knees^6^.

One of the reasons near-normal kinematics are observed in UKA knees may be due to the preservation of the anterior cruciate ligament (ACL) and posterior cruciate ligament (PCL) during this procedure. In normal knees, the ACL is shortened with flexion, while the PCL extends during flexion^8,9^. In contrast, several studies have demonstrated that the cruciate ligament force following BCR-TKA was higher than that of normal knees^10,11^. Moreover, the postoperative ACL force was higher than that observed preoperatively. However, the cruciate ligament force of UKA knees remains unknown.

Several studies have reported that the knee kinematics among patients with OA are different from normal knee kinematics^12,13^. Therefore, a patient’s preoperative osteoarthritic condition might affect postoperative knee kinematics. However, the association between kinematics and cruciate ligament force before and after UKA is largely unknown. Moreover, the independent relation between kinematics and the cruciate ligament force remains unknown as well.

Numerous people perform high knee flexion activities, such as gardening or exercising. Several studies have reported that participating in high knee flexion activities is related to better clinical outcome, patient satisfaction, and patient expectations after knee joint replacement^14,15^. Therefore, we aimed to evaluate high knee flexion activities within the scope of this study.

This study was designed to analyze the effects of implantation on the cruciate ligament force in UKA and to determine whether kinematics is associated with the cruciate ligament force. We hypothesized that implantation would affect and increase the cruciate ligament force. This is because there is a significant difference in the varus–valgus angulation between before and after UKA, and a previous study has demonstrated cruciate ligament elongation after coronal realignment in TKA^16^.

## Results

### Kinematic changes

The preoperative and postoperative knees were flexed from 4.1 ± 7.4° to 134.9 ± 14.3°, and from 3.8 ± 6.0° to 129.1 ± 15.2°, respectively, on average. There were no significant differences in the minimum and maximum flexion angle between the preoperative and postoperative values (p = 0.11 and 0.89 in the minimum and maximum flexion, respectively).

**Figure 1:**
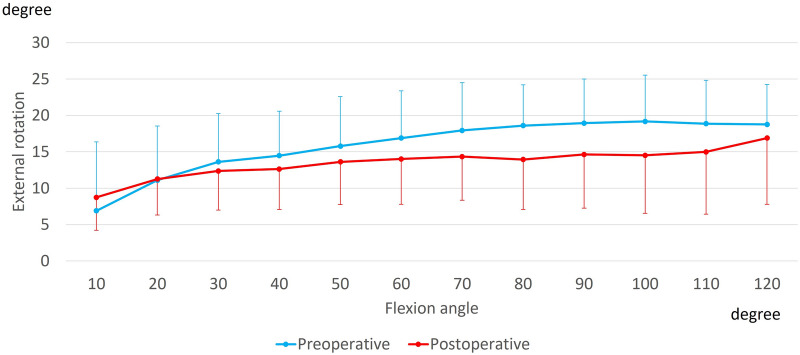
Rotation angle during squatting. The femur displayed external rotation with flexion.

Preoperatively, the femurs displayed steep external rotation (8.0 ± 5.1°) relative to the tibia from 10° to 50° of flexion, reaching 19.5 ± 4.2° on average. Postoperatively, the femurs displayed steep external rotation (4.9 ± 4.2°) relative to the tibia from 10° to 50° of flexion, reaching 17.3 ± 6.9° on average. There were no significant differences between the preoperative and postoperative knees (p = 0.48) (Fig. 1).

**Figure 2:**
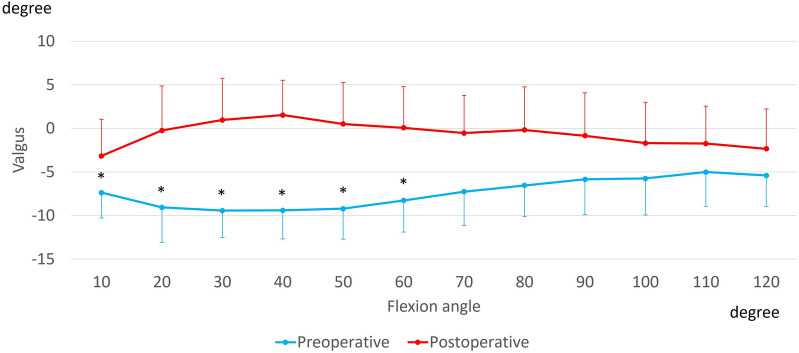
Varus–valgus angle during squatting. *Significant differences between preoperative and postoperative knees (*p* ≤ 0.05).

The preoperative knees showed 1.4 ± 4.1° varus movement up to 50° of flexion, while the postoperative knees showed no significant movement. From 10° to 60° of flexion, the preoperative knees indicated a significant varus position compared to the postoperative knees (Fig. 2).

**Figure 3:**
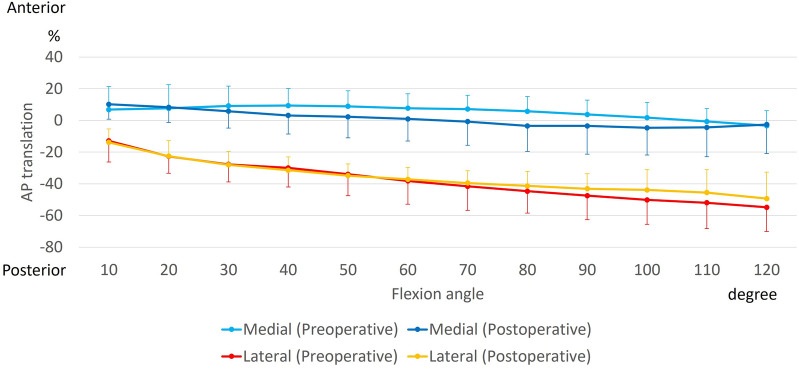
Anteroposterior (AP) translation of medial and lateral side of the femur during squatting. AP translation is calculated as a percentage relative to AP length of the tibia.

The preoperative and postoperative AP translation of the medial side indicated 10.8 ± 16.0% and 18.1 ± 12.1% posterior movement with flexion, respectively. There was no significant difference between the preoperative and postoperative values (p = 0.44) (Fig. 3).

The preoperative and postoperative AP translation of the lateral side indicated 46.7 ± 20.2% and 41.7 ± 19.3% posterior movement with flexion, respectively. There was no significant difference between the preoperative and postoperative values (p = 0.28) (Fig. 3).

### ACL forces

**Figure 4:**
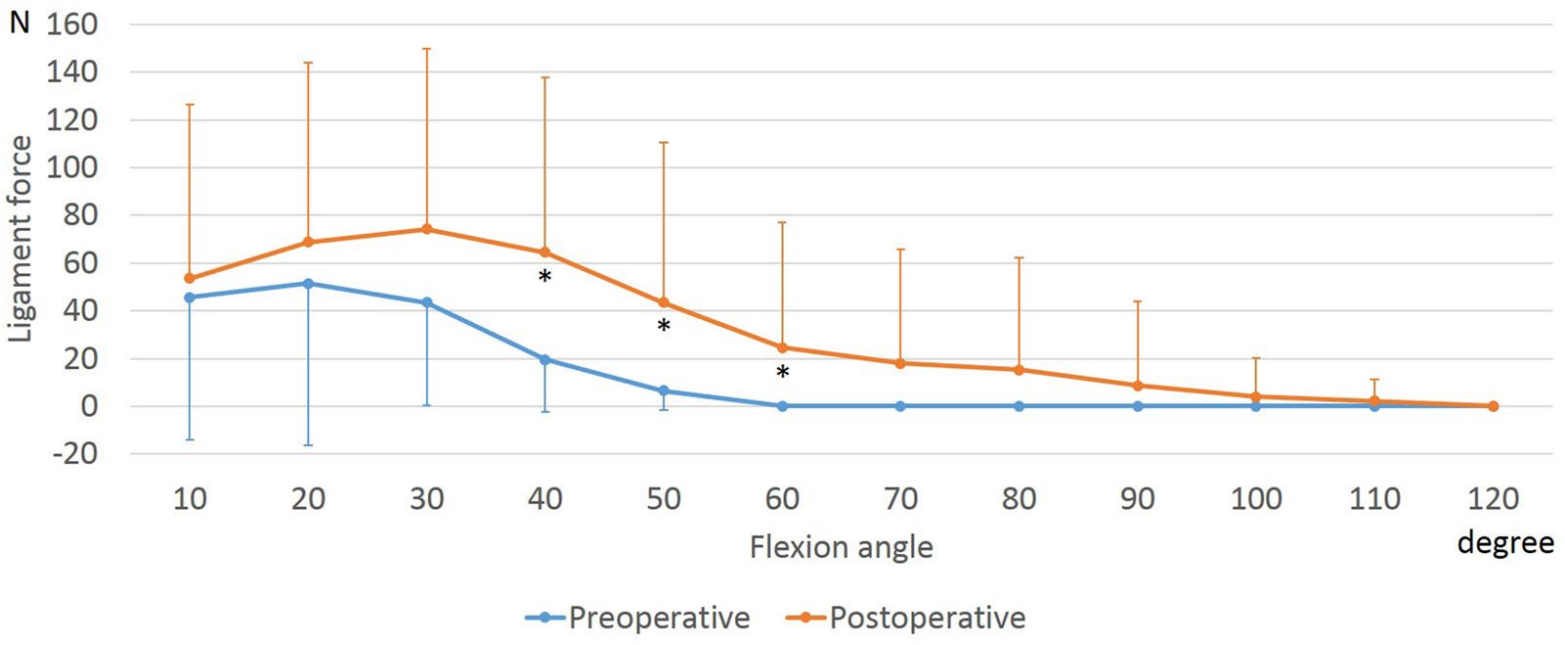
Force of the anteromedial bundle of the anterior cruciate ligament during squatting before and after unicompartmental knee arthroplasty. *Significant differences between preoperative and postoperative knees (*p* ≤ 0.05).

**Figure 5:**
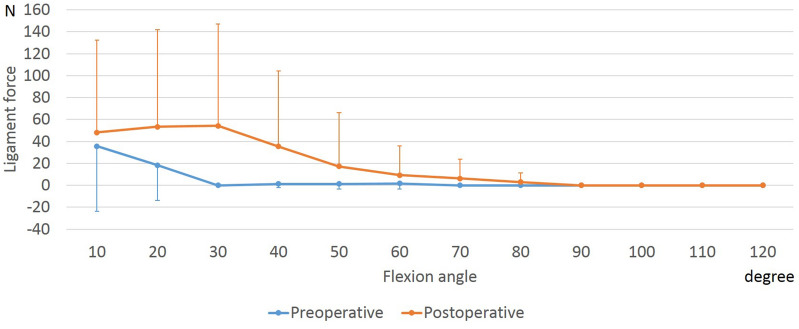
Force of the posterolateral bundle of the anterior cruciate ligament during squatting before and after unicompartmental knee arthroplasty.

The femoral and tibial attachment areas of the anteromedial and posterolateral bundles of the ACL (aACL and pACL) decreased with flexion both preoperatively and postoperatively (Figs. 4, 5). At 40° to 60° of flexion, the postoperative aACL force was larger than the preoperative one.

### PCL forces

**Figure 6:**
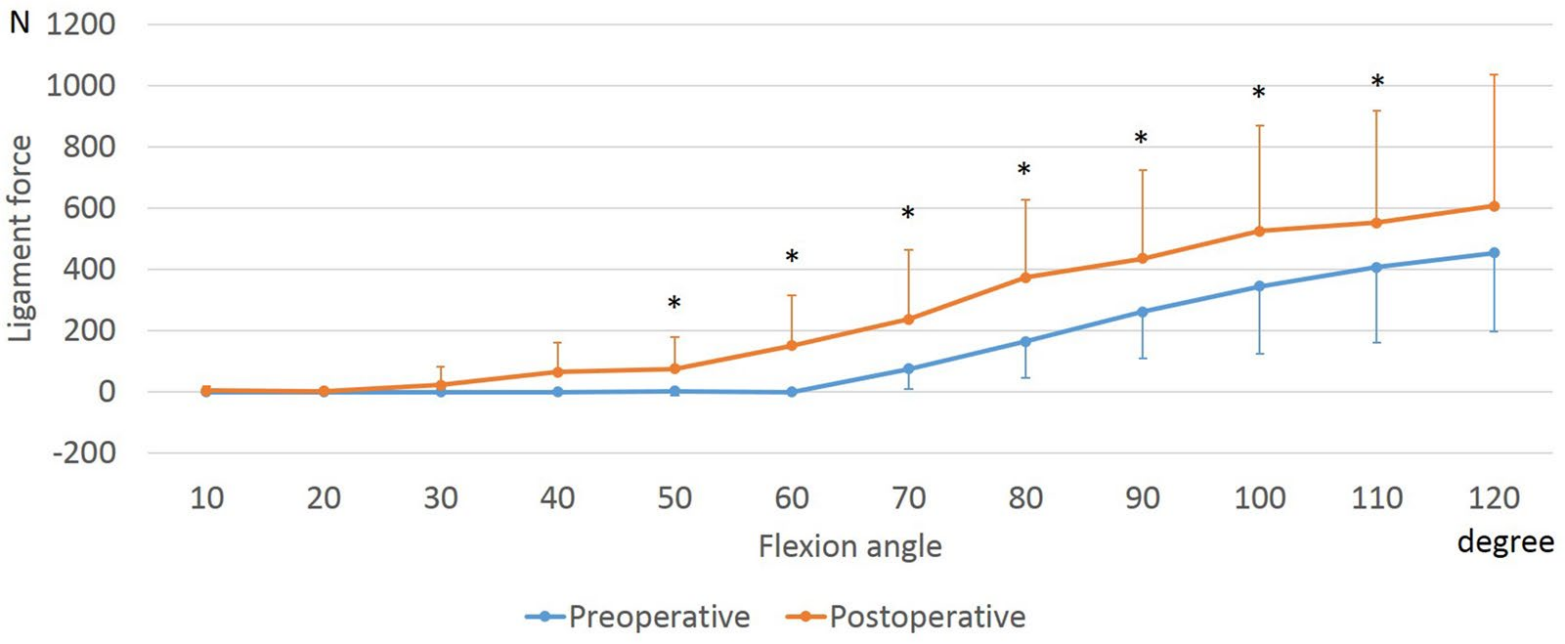
Force of the anterolateral bundle of the posterior cruciate ligament during squatting before and after unicompartmental knee arthroplasty. *Significant differences between preoperative and postoperative knees (*p* ≤ 0.05).

**Figure 7:**
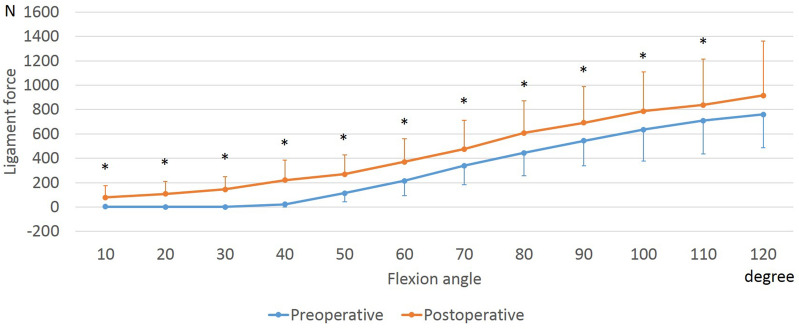
Force of the posteromedial bundle of the posterior cruciate ligament during squatting before and after unicompartmental knee arthroplasty. *Significant differences between preoperative and postoperative knees (*p* ≤ 0.05).

The anterolateral and posteromedial bundles of the PCL (aPCL and pPCL) increased with flexion both preoperatively and postoperatively (Figs. 6, 7). At 50° to 110° of flexion, the postoperative aPCL force was larger than the preoperative one. In addition, at 10° to 110° of flexion, the postoperative pPCL force was larger than the preoperative one.

### Correlation between kinematics and the cruciate ligament force

Linear regression was used to determine whether knee kinematic patterns were associated with ligament tensile forces (Table 1). Preoperatively, the aACL forces were highly correlated with the AP translation of the lateral condyles. In addition, the pPCL forces were highly correlated with the varus–valgus angulation. However, postoperatively, the PCL forces in both bundles were highly correlated with the AP translation of the medial femoral condyle.

**Table 1:** Correlation between differences in knee kinematics and cruciate ligament.

	Preoperative		Postoperative	
	Correlation coefficient	p-value	Correlation coefficient	p-value
**aACL**				
Rotation	0.21	0.48	0.23	0.45
Varus–valgus	0.30	0.30	0.61	0.03
Medial AP translation	0.37	0.20	0.33	0.27
Lateral AP translation	0.59	0.03	0.24	0.42
**pACL**				
Rotation	− 0.01	0.98	− 0.03	0.92
Varus–valgus	0.41	0.15	0.12	0.70
Medial AP translation	0.13	0.66	− 0.51	0.08
Lateral AP translation	0.44	0.12	− 0.50	0.08
**aPCL**				
Rotation	0.09	0.76	0.05	0.87
Varus–valgus	− 0.35	0.21	0.36	0.22
Medial AP translation	0.11	0.71	0.62	0.02
Lateral AP translation	− 0.23	0.43	0.54	0.06
**pPCL**				
Rotation	0.21	0.47	− 0.04	0.89
Varus–valgus	− 0.57	0.03	0.34	0.25
Medial AP translation	0.33	0.25	0.60	0.03
Lateral AP translation	− 0.18	0.53	0.55	0.05

*aACL* anteromedial bundle of the anterior cruciate ligament, *aPCL* posterior bundle of the anterior cruciate ligament, *AP* anteroposterior, *pACL* anterolateral bundle of the posterior cruciate ligament, *pPCL* posteromedial bundle of the posterior cruciate ligament.

## Discussion

The most important findings of this study were that the knees of the patients with OA showed a larger cruciate ligament force following UKA than that before UKA, and the cruciate ligament force was correlated with the varus–valgus angulation and AP translation.

Mochizuki et al. have reported that UKA does not completely recreate normal knee kinematics^7^. However, a previous study has demonstrated that the kinematics following UKA are more similar to the normal knee kinematics than those following BCR-TKA^5^. Furthermore, the kinematics before UKA resembled those following UKA in terms of axial rotation and AP translation; although the preoperative knees indicated a significant varus alignment compared with the postoperative knees^6^. In this study, the postoperative ACL and PCL forces were larger than the preoperative values. These facts suggest that coronal realignment drives the re-tensioning of cruciate ligaments.

Valgus angulation was positively correlated with the aACL force during postoperative squatting; however, it was negatively correlated with the pPCL force during preoperative squatting. This suggests that valgus angulation with flexion postoperatively increases the aACL force, and severe varus angulation preoperatively increases the pPCL. Specifically, the excessive postoperative valgus angulation might result in a non-physiological ligament force. Hopgood et al. have reported that a 2-mm increase in insert thickness achieved a correction of 1.5°–2.9°^17^. Further, Walker et al. have reported that updated instrumentation appears to be an effective tool in determining an adequate level of tibial resection, which, in turn, prevents unnecessary bone loss and reduces the need for thicker bearings^18^. The bearing thickness in this study was 4.0 ± 0.9 mm. Therefore, the bearing thickness might affect the coronal alignment and cruciate ligament force. This is because such a large bearing thickness might drive the valgus angulation and subsequently increase the aACL force.

The lateral AP translation was positively correlated with the aACL force during preoperative squatting. The medial and lateral AP translations were positively correlated with both the aPCL and pPCL forces during postoperative squatting. This suggests that reduced posterior translation on the lateral femoral condyle induces ACL tightness preoperatively, and reduced femoral rollback from mid-flexion to high-flexion induces PCL tightness postoperatively.

According to a study, the ACL force in the postoperative knees was greater than the force in the preoperative knees following BCR-TKA; this is similar to our present findings^19^. Okada et al. demonstrated that the in situ ACL force against 100 N of anterior force in BCR-TKA knees was statistically comparable with that of intact knees at all flexion angles^10^. Furthermore, Sabouret et al. have reported that TKA with retention of the ACL remained functional and provided adequate stability at 22 years of follow-up^20^. In TKA, sacrificing the ACL reportedly achieved inferior clinical outcomes^21^. Moreover, compared to posterior cruciate-retaining (PCR)-TKA, BCR-TKA achieved more normal-like kinematics^22^. Finally, it has been reported that normal-like kinematics promote favorable clinical outcomes^23^. These facts suggest that ACL retention in UKA drives a strongly favorable clinical outcome.

Although there was no significant difference in the PCL force in BCR-TKA^19^, the PCL force in the postoperative UKA knees was larger than that in the preoperative knees. Tsai et al. have reported that the PCL in BCR-TKA was significantly overstretched in deep flexion positions, resonating with a previous posterior cruciate-retaining TKA study^16^, which reported overstretching of the PCL in PCR-TKA during deep flexion and attributed this to reduced femoral rollback^24^. The PCL in UKA might be overstretched. In addition, overstretching of the PCL might increase the ligament force. However, the PCL tension in UKA may be larger than that in TKA, even though the PCL in both UKA and TKA is elongated.

Malalignment of the lower limb following UKA is associated with a poorer clinical outcome^25^. Since no ligament release is performed in UKA, a precise osteotomy could result in preservation of the appropriate ligament balance. Several previous studies have reported that navigation- and robotic-assisted UKAs improve the accuracy and survival following surgery^26,27^. To improve osteotomy accuracy and for achieving appropriate ligament balance, a widespread use of navigation- and robotic-assisted UKAs may be important.

This study has some limitations. First, we did not evaluate normal knees since there were no patients with non-arthritic contralateral knees included in the study. Therefore, the difference between UKA knees and normal knees remains unclear. Second, this study cohort had a relatively short mean follow-up duration of 9.5 months. The kinematics and ligament force at long-term follow-up may differ from those reported in this study. Third, the relationship between soft tissue balance and cruciate ligament force remains unclear since the gap was evaluated based on a feel-gauge rather than quantitatively. Fourth, in this study, only mobile-bearing UKA was analyzed. Several studies have reported that the kinematics in mobile-bearing UKA is different from that of fixed-bearing UKA^6,28^. Therefore, our findings may not be representative of fixed-bearing UKA. Fifth, only patients with OA were included in the study. Therefore, the findings may not be representative of patients with osteonecrosis. Sixth, only patients who could perform squatting activities were included in the current study; therefore, our findings may not be generalizable to those who cannot perform such activities.

In conclusion, the ACL and PCL forces of the knees following UKA were larger than those of the knees before UKA. Moreover, the kinematic changes were significantly correlated with changes in the cruciate ligament forces.

## Methods

We investigated a total of 16 patients (17 knees) who underwent medial UKA (Oxford partial knee, Zimmer Biomet G.K., Warsaw, USA). Patients who suffered from medial OA but could perform squatting activities were included. The patients provided informed consent to participate in this investigation, which was approved by the institutional review board (provided by The University of Tokyo Institutional Ethics Review Board).

The following methods were carried out in accordance with the relevant guidelines and regulations. All surgeries were performed using a minimally invasive approach to comply with the Oxford Group recommendations^29,30^. Using a sagittal saw blade aimed towards the hip center, a vertical tibial incision was made at the medial edge of the anterior cruciate ligament insertion on the tibia. Subsequently, a horizontal incision was made using the tibial saw guide, which had a 7° built-in posterior slope set parallel to the long axis of the tibia in the coronal and sagittal planes. Femoral drilling was performed using an Oxford Microplasty device (MP: Biomet Ltd., Swindon, UK) to facilitate reproducible implantation^18^. Following these procedures, we performed a gap balancing procedure between knee flexion and extension and a modified keel cutting method as previously reported^31^. With the trial component and bearing in place, the knee was manipulated through a full range-of-motion (ROM) to demonstrate joint stability, security of the mobile-bearing joint, and absence of impingement.

The surgeries were performed by five knee surgeons, and a highly experienced surgeon (TT or HI) oversaw all procedures as either the chief surgeon or first assistant.

Each patient was asked to perform a squatting activity. Fluoroscopic surveillance was performed in the sagittal plane while each patient performed a squatting motion at a natural pace. The patients practiced the motion several times before the recording. Knee motions were recorded before (within 1 month) and after (at least 6 months) the UKA. At the postoperative fluoroscopic analysis, the patients’ mean age was 73.2 ± 6.5 years. The patients’ mean body height was 154.6 ± 9.7 cm and their mean body weight was 57.9 ± 7.7 kg. The mean duration of postoperative follow-up was 9.5 ± 2.3 months. Among the 16 patients (17 knees) included in the analysis, 6 were men and 11 were women. All patients underwent UKA to treat medial knee joint OA (Kellgren and Lawrence grade III). The mean hip–knee–ankle angles at the time of analysis were 172.6° ± 3.1° preoperatively and 174.8° ± 3.1° postoperatively^6^.

The sequential motion was recorded as digital X-ray images (1024 × 1024 × 12 bits/pixel, 7.5-Hz serial spot images as a DICOM file) using a 17-inch flat panel detector system (C-vision Safire L, Shimadzu, Kyoto, Japan and ZEXIRA DREX-ZX80, Toshiba, Tokyo, Japan). All images were processed using dynamic range compression to enable edge-enhanced images. To estimate the spatial position and orientation of the knee, a two-dimensional (2D)/three-dimensional (3D) registration technique was used^32,33^. This technique is based on a contour-based registration algorithm that uses single-view fluoroscopic images and 3D computer-aided design (CAD) models^32,33^. We created 3D bone models using computed tomography (CT) before surgery and used them for CAD models. The estimation accuracy for relative motion between 3D bone models was ≤ 1° in rotation and ≤ 1 mm in translation^33^. The local coordinate system in the bone model was produced according to a previous study^33^. Knee rotations were described using the joint rotational convention of Grood and Suntay^34^. The femoral rotation and varus–valgus angle relative to the tibia, and the AP translation of the medial sulcus (medial side) and lateral epicondyle (lateral side) of the femur on the plane perpendicular to the tibial mechanical axis during each flexion angle were evaluated^6,33^. AP translation was calculated as a percentage relative to the proximal AP dimension of the tibia^6,33^. External rotation was denoted as positive, and internal rotation as negative. Valgus was defined as positive, and varus as negative. Positive or negative values of AP translation were defined as anterior or posterior to the axis of the tibia, respectively.

Both bundles of the ACL and PCL were identified using the osseous landmarks on the preoperative CT and magnetic resonance imaging (MRI)^8,35–37^. The accuracy of the attachment area was within 0.7 ± 0.1 mm^37^. Each cruciate ligament force was calculated using commercially available software (VivoSim, Advanced Mechanical Technology Inc., Watertown, MA, USA). The path of each ligament was assumed to be a straight line, and the effects of the ligament-bone contact were neglected. Each ligament was assumed to be elastic, and its properties were described using a nonlinear force–strain curve^38–40^. The stiffness values and reference lengths of the model ligaments were based on the data reported by Shelburne et al.^40,41^; the properties of the model ligaments were adjusted to match the measurements of the intact knee-joint laxity and ACL-deficient knees obtained from previous cadaver studies^39,42^. The ligament stiffnesses assumed in the model are shown in Table 2. The cruciate ligament forces at each flexion were evaluated (Video 1). To investigate whether ligament forces were related to the knee kinematics, we computed the changes in femoral rotation, varus–valgus angulation, and AP position of the femoral condyles, as well as changes in ligament forces for each ligament bundle from 10° to 120° of flexion^6^. We then evaluated the correlation between the change in each kinematic parameter and the change in force for each cruciate ligament bundle. AP translation was calculated as a percentage of the proximal AP dimension of the tibia^6^. External rotation was denoted as positive, and internal rotation was denoted as negative. Valgus angulation was defined as positive, whereas varus was defined as negative. Positive or negative values of AP translation were defined as anterior or posterior to the axis of the tibia, respectively. All values were expressed as means ± standard deviations.

**Table 2:** Values of knee ligament stiffnesses assumed in the model.

Ligament	Stiffness (N/strain)
aACL	1000
pACL	1500
aPCL	2600
pPCL	1900

*aACL* anteromedial bundle of the anterior cruciate ligament, *aPCL* posterior bundle of the anterior cruciate ligament, *pACL* anterolateral bundle of the posterior cruciate ligament, *pPCL* posteromedial bundle of the posterior cruciate ligament.

### Statistical analyses

The results were analyzed using IBM Corp. (Released 2017. IBM SPSS Statistics for Windows, Version 25.0. Armonk, NY: IBM Corp). Repeated-measures analysis of variance (ANOVA) and post-hoc pairwise comparison (Bonferroni test) were used to analyze all evaluation items and compare for multiple comparisons. Pearson’s correlation coefficient was used to analyze the correlation between differences in knee kinematics and the corresponding differences in cruciate ligament force. Statistical significance was set at *p* ≤ 0.05. Moreover, a power analysis using Easy R (EZR)^43^ indicated that a dataset with data from nine knees would be required for an alpha set at 0.05, and statistical power set at 0.8.

## Supplementary Information

Supplementary Video 1.

## Data Availability

The datasets generated during and/or analyzed during the current study are available from the corresponding author on reasonable request.
